# An Infancy-Onset 20-Year Dietary Counselling Intervention and Gut Microbiota Composition in Adulthood

**DOI:** 10.3390/nu14132667

**Published:** 2022-06-27

**Authors:** Anniina Keskitalo, Eveliina Munukka, Anna Aatsinki, Wisam Saleem, Noora Kartiosuo, Leo Lahti, Pentti Huovinen, Laura L. Elo, Sami Pietilä, Suvi P. Rovio, Harri Niinikoski, Jorma Viikari, Tapani Rönnemaa, Hanna Lagström, Antti Jula, Olli Raitakari, Katja Pahkala

**Affiliations:** 1 Research Centre of Applied and Preventive Cardiovascular Medicine, University of Turku, 20520 Turku, Finland; anniina.johanna.keskitalo@tyks.fi (A.K.); noora.kartiosuo@utu.fi (N.K.); suvi.rovio@utu.fi (S.P.R.); harri.niinikoski@utu.fi (H.N.); olli.raitakari@utu.fi (O.R.); 2 Centre for Population Health Research, University of Turku and Turku University Hospital, 20520 Turku, Finland; anna.a.aatsinki@utu.fi (A.A.); hanna.lagstrom@utu.fi (H.L.); 3 Department of Clinical Microbiology, Turku University Hospital, 20520 Turku, Finland; penhuo@utu.fi; 4 Microbiome Biobank, Institute of Biomedicine, University of Turku, 20520 Turku, Finland; laevmu@utu.fi; 5 Department of Computing, Faculty of Technology, University of Turku, 20520 Turku, Finland; wisam.tariqsaleem@utu.fi (W.S.); leo.lahti@utu.fi (L.L.); 6 Department of Mathematics and Statistics, University of Turku, 20520 Turku, Finland; 7 Institute of Biomedicine, University of Turku, 20520 Turku, Finland; laura.elo@utu.fi; 8 Turku Bioscience Centre, University of Turku and Åbo Akademi University, 20520 Turku, Finland; sami.pietila@utu.fi; 9 Department of Physiology/Department of Pediatrics, University of Turku, 20520 Turku, Finland; 10 Department of Medicine, University of Turku, 20520 Turku, Finland; jorma.viikari@utu.fi (J.V.); tapani.ronnemaa@utu.fi (T.R.); 11 Division of Medicine, Turku University Hospital, 20520 Turku, Finland; 12 Department of Public Health, University of Turku and Turku University Hospital, 20520 Turku, Finland; 13 Department of Public Health Solutions, Institute for Health and Welfare, 20520 Turku, Finland; antti.jula@fimnet.fi; 14 Department of Clinical Physiology and Nuclear Medicine, Turku University Hospital, University of Turku, 20520 Turku, Finland; 15 Paavo Nurmi Centre & Unit for Health and Physical Activity, University of Turku, 20520 Turku, Finland

**Keywords:** dietary counselling, low-saturated fat diet, gut microbiota, microbiota diversity

## Abstract

The randomized controlled Special Turku Coronary Risk Factor Intervention Project (STRIP) has completed a 20-year infancy-onset dietary counselling intervention to reduce exposure to atherosclerotic cardiovascular disease risk factors via promotion of a heart-healthy diet. The counselling on, e.g., low intake of saturated fat and cholesterol and promotion of fruit, vegetable, and whole-grain consumption has affected the dietary characteristics of the intervention participants. By leveraging this unique cohort, we further investigated whether this long-term dietary intervention affected the gut microbiota bacterial profile six years after the intervention ceased. Our sub-study comprised 357 individuals aged 26 years (intervention *n* = 174, control *n* = 183), whose gut microbiota were profiled using 16S rRNA amplicon sequencing. We observed no differences in microbiota profiles between the intervention and control groups. However, out of the 77 detected microbial genera, the *Veillonella* genus was more abundant in the intervention group compared to the controls (log_2_ fold-change 1.58, *p* < 0.001) after adjusting for multiple comparison. In addition, an association between the study group and overall gut microbiota profile was found only in males. The subtle differences in gut microbiota abundances observed in this unique intervention setting suggest that long-term dietary counselling reflecting dietary guidelines may be associated with alterations in gut microbiota.

## 1. Introduction

The gut microbiome is suggested to play a vital part in human health and physiology [1,2,3]. While the human gut provides an appropriate living environment for microbes, these microbes in turn contribute to, e.g., host energy metabolism and innate immunity. Gut microbes may, for example, utilize dietary components that are indigestible for host enzymes, and produce a vast number of metabolites that can serve as signaling molecules and/or energy supplies for the host [1]. If the balance of the intestinal microbiota is perturbed, the metabolic and regulatory activities of the microbes can become harmful for the host and promote inflammation or metabolic disorders [4,5].

One of the key factors determining the gut microbiota is diet [6,7]. Adult gut microbiota are relatively stable and largely determined by long-term dietary patterns [8,9]; yet, a diet switch can revise the composition and metabolic functions of gut microbes [8,10]. Dietary protein, fat, and carbohydrates have all been reported to modify the intestinal microbiota, however, the exact role of different food constituents in maintaining a healthy gut environment and promoting the growth of beneficial gut bacteria remain largely undefined [6,11,12]. Regarding the effects of diet on gut microbiota, dietary fiber is the most extensively studied food constituent. Dietary fibers serve as substances for bacterial fermentation, and therefore significantly affect gut microbiota composition and activity [6]. A higher intake of dietary fiber has been associated with improved health outcomes such as a reduced risk of cardiovascular diseases (CVD), type 2 diabetes (T2D), obesity, and cancer, which may at least partially be linked to increased short-chain fatty acid production via gut microbial fermentation [13]. In contrast, a Western diet (generally low in fiber and high in saturated fat (SAFA)), is suggested to disrupt intestinal homeostasis and promote inflammation [9,14]. In mice, the type of dietary fatty acids, i.e., SAFA vs. unsaturated fat, has been shown to profoundly impact gut microbial composition and diversity [15,16]. Moreover, a diet high in SAFA appears to promote inflammation, partially due to interaction with gut microbes [15], while the ingestion of omega-3 polyunsaturated fatty acids (PUFA) apparently tends to increase the abundance of certain gut bacteria that are generally regarded as beneficial, such as *Bifidobacterium* spp. and *Lactobacillus* spp. [15,17]. Based on observational studies, high SAFA intake can lead to changes in the gut microbiome that are associated with an unhealthy metabolic state (reviewed in [18]). A small randomized trial has, consistently with the aforementioned mouse studies, reported a reversible increase in *Bifidobacterium* and *Lactobacillus* genera during an eight-week omega-3 PUFA intervention [12]. However, other supplementation trials have failed to observe associations between PUFA intake and gut microbiota [19,20]. Despite the knowledge gained through these studies on the links between diet and gut microbiota, long-term interventions focusing on the effects of dietary fatty acids and prevention of CVD in humans remain scarce.

The Special Turku Coronary Risk Factor Intervention Project (STRIP) is a unique long-term dietary counselling intervention experiment; for the past three decades, it has explored the effects of infancy-onset dietary counselling on cardiometabolic health [21]. By repeated dietary counselling from the age of 7 months to 20 years, the intervention group was introduced to a heart-healthy diet, characterized especially by low proportional intake of SAFA and cholesterol. This repeated dietary counselling reportedly reduced the intake of SAFA and increased the intake of PUFA and fiber during the intervention period [22,23]. The STRIP intervention effects have been largely maintained into adulthood six years after the withdrawal of the intervention [24]. As dietary counselling introduced in infancy and continued throughout childhood and adolescence appears to improve the diet quality into adulthood, the intervention effects may be reflected in the intestinal microbiota of the participants. Therefore, this study aimed to investigate whether the infancy-onset 20-year dietary counselling intervention involving a more heart-healthy diet in the intervention participants affected their gut microbiota bacterial profile in adulthood.

## 2. Materials and Methods

### 2.1. Study Design

**Figure 1:**
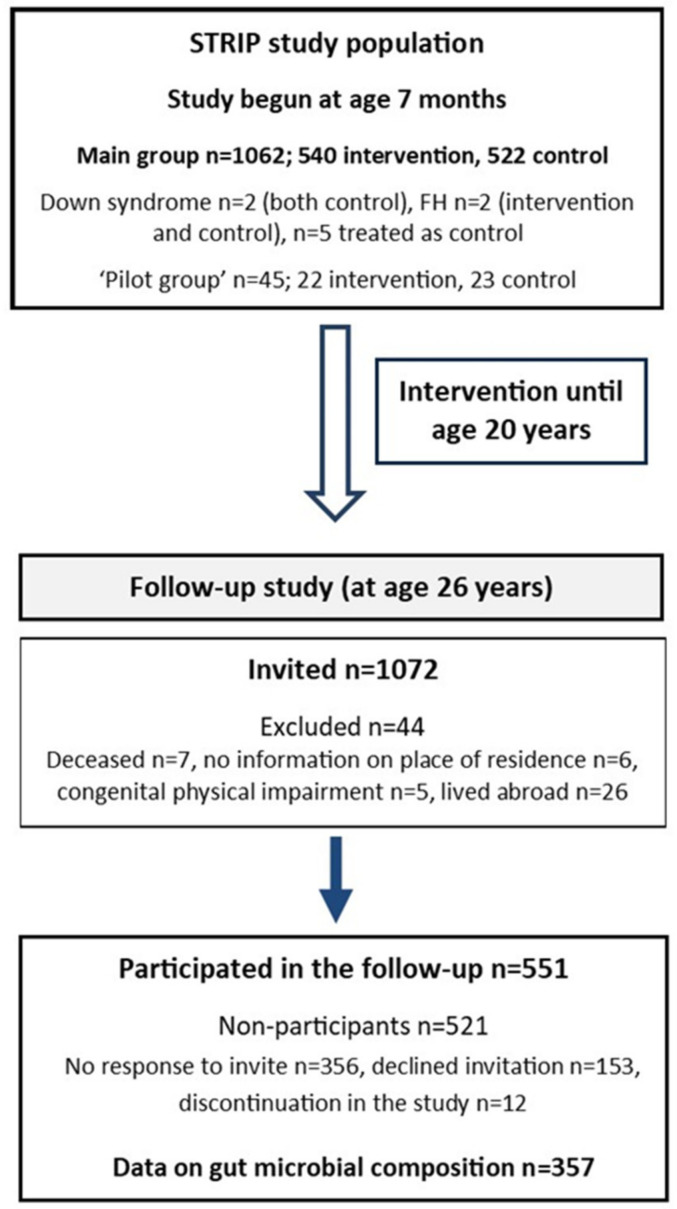
Flow chart of the STRIP study (FH; familial hypercholesterolemia).

The STRIP study is a prospective randomized trial which aims to prevent atherosclerosis beginning in infancy [21]. In brief, families of 5-month-old infants born between July 1989 and December 1991 were recruited at well-baby clinics in Turku, Finland by nurses. At the age of 7 months, 1062 infants (56.5% of the eligible age-cohort) were randomly allocated to a dietary intervention (*n* = 540) or control (*n* = 522) group (Figure 1). The cohort included two children with Down syndrome (both control), two with familial hypercholesterolemia (intervention and control), and five children who had been randomized to the intervention group, and who missed the first study visits prior to age 13 months, and were thus later treated as controls. Additionally, a group of 45 children born between March and July 1989 was similarly recruited and randomized (intervention *n* = 22, control *n* = 23) to first test the study protocols, and thus served as a ‘pilot’ group.

The intervention group received individualized dietary counselling at 1- to 3-month intervals until age 2 years, and biannually thereafter until the age of 20 years [22]. The counselling was provided to parents until the child was aged seven years, and thereafter more information was gradually provided directly to the child. The intervention consisted of 30-minute individualized counselling sessions led by a nutritionist. Each session had a specific dietary topic and involved performing various tasks. The parents were informed of the sessions’ topics and tasks and encouraged to discuss them at home. Furthermore, parents/children received oral and written feedback about their child’s diet. The main target of the counselling was to replace SAFA with unsaturated fat in the child’s diet and concomitantly reduce the intake of cholesterol; a reduction in total fat intake was not targeted. The intervention group received counselling on how to reduce salt intake and to favour wholegrain products instead of more highly refined options. The counselling further encouraged the inclusion of fruits, vegetables, and berries in the diet. A fixed diet was never specified; the counselling was individualized and the child’s recent food diary was used as a basis for suggested dietary changes. The dietary recommendations were based on the latest available version of the Nordic nutrition recommendations (e.g., 30% of energy intake (E%) from fat, <10 E% from SAFA, 10–15 E% from protein, and 50–60 E% from carbohydrates). As part of the intervention, primary prevention of smoking was introduced at age eight years. This was based on supporting the self-image of non-smoking children and on understanding the health risks associated with both active and passive smoking. A physically active lifestyle was encouraged, although it was not a structured, continuous part of the intervention.

The children in the control group did not receive the counselling intervention, although similar measurements, including keeping of food diaries, were performed for both study groups and they met with the same study personnel. The control children were seen biannually until the age of seven years and annually thereafter.

The first post-intervention follow-up with the participants was conducted between April 2015 and January 2018 at the age of 26 years, six years after the intervention had ended [24] (Figure 1). Of the participating cohort (*n* = 1116), 1072 were invited to participate (excluded, *n* = 44; deceased, *n* = 7; no information on place of residence, *n* = 6; congenital physical impairment, *n* = 5; lived abroad, *n* = 26). Of these, 551 provided follow-up data (51%; intervention, *n* = 263 vs. control, *n* = 288). More females attended the follow-up than males (*n* = 308 vs. *n* = 243). Of the participants, only five provided questionnaire data who did not participate in the clinic visit. Reasons for non-participation (*n* = 521) were: no response to invite (*n* = 356); declined invitation (*n* = 153); and discontinuation of the study (*n* = 12). Individuals included in the present study comprise those who provided data on gut microbiota composition at the 26-year follow-up (*n* = 357).

The STRIP study has been approved by the associated university and hospital district ethical authorities. Written informed consent was obtained from parents at study entry and from the participants at the ages of 15, 18, and 26 years.

### 2.2. Fecal Microbiota Composition

The gut microbiota of the STRIP participants was assessed for the first time in the 26-year follow-up study. Fecal samples were collected by the participants at their homes and sent to the study center by mail (*n* = 370). A pre-filled mail package, including self-collection equipment and instructions, was provided at the study visit. In addition, a sample form inquiring about stool composition (e.g., diarrhea, constipation) during the past week, history of gastroenteritis (past three months), use of antibiotics (past three months), and use of probiotics (past month) was provided to the subjects to be filled out at the time of sampling and returned together with the sample.

The participants were guided to collect a small amount (approximately 500 mg) of fecal material in an OMNIgene^®^ GUT collection tube (DNA Genotek, Ottawa, ON, Canada), to homogenize the sample by vigorous shaking for 30 s, to mark the date and time of sampling on the accompanying sample form, and to pack and mail the sample and sample form to the laboratory as soon as possible after the collection. OMNIgene^®^ GUT collection tubes include a stabilizing solution that guarantees DNA integrity in typical ambient temperature fluctuations and stability at room temperature for as long as 60 days, thus enabling shipping and storage at ambient temperatures.

In the laboratory, three samples were omitted due to poor sample quality. The samples were homogenized by gentle mixing, and bacterial DNA was extracted from 200–250 µL of sample solution with a GXT Stool Extraction Kit VER 2.0 (Hain Lifescience GmbH, Nehren, Germany). The extraction was otherwise performed according to the manufacturer’s instructions, although sample vortexing was replaced by homogenization with a MOBIO PowerLyzer 24 Bench Top Bead-Based Homogenizer in 1.4 mm ceramic bead tubes (MO BIO Laboratories, Inc., Carlsbad, CA, USA) at 1000 rpm for 3 min to induce cell lysis. The DNA concentrations were measured with a Qubit dsDNA HS Assay kit and Qubit 2.0 fluorometer (Thermo Fisher Scientific, Waltham, MA, USA), and DNA was stored at −75 °C. Fecal microbiota profiles were analyzed by 16S rRNA gene sequencing; variable region V4 of the bacterial 16S rRNA gene was amplified with custom-designed dual-indexed primers and sequenced with an Illumina MiSeq system as previously described [25]. Each sequencing run included a positive plasmid-mix control and a negative aqua control.

The raw 16S rRNA gene sequencing data were demultiplexed and the sequence adapters, primers, and barcodes were clipped using the Illumina BaseSpace platform. Ten samples were excluded from further analyses due to unsuccessful 16S rRNA gene sequencing, resulting in a final sample cohort of 357 individuals. The raw sequence data were processed into an amplicon sequence variant (ASV) table using the *DADA2* pipeline [26]. First, the demultiplexed fastq files were filtered and trimmed, each sample was dereplicated, and a portion of the data set was used to estimate the error parameters. Then, function dada was applied using the inferred error parameters and chimeric sequences were filtered out using function isBimeraDenovo. The generated ASV table altogether comprised 6.3 × 10^7^ trimmed and chimera-removed high-quality sequence reads. The acquired read counts from the 16S rRNA gene sequencing varied significantly within the study population (min: 11.8 k, max: 839 k, median: 160 k). The total read counts were similar in the control (min: 19.7 k, max: 839 k, median: 165 k) compared to the intervention group (min: 11.8 k, max: 752 k, median: 157 k) (Kruskal–Wallis *p* = 0.09).

Taxonomic classification of the sequences was performed using the NCBI RefSeq 16S rRNA database supplemented by the Ribosomal Database Project database (RefSeq-RDP16S_v2_May2018). The generated unfiltered phyloseq object altogether included 6591 unique ASVs that corresponded to 20 different bacterial phyla and 291 bacterial genera. A sequencing batch effect (*n* = 5) was detected related to beta diversity and dispersion. Differences in beta diversity analyzed with PERMANOVA implemented in the adonis function of the *vegan* package showed a nominal difference according to batch (R^2^ = 0.018, *p*-value = 0.02). However, this effect may have been influenced by beta dispersion (PERMDISP2 implemented in betadisper in *vegan* package, ANOVA *p* = 0.027). Bray–Curtis dissimilarity was calculated based on rarefied ASV abundance matrix. The intervention and control participants were distributed equally across the batches (batches 1 to 5, intervention/control: 38/62%, 47/53%, 49/51%, 51/49%, 57/43%, respectively; χ^2^ *p* = 0.22). There was a nominal sex difference in the batches (batches 1 to 5, female/male: 70/30%, 52/48%, 43/57%, 55/45%, 62/38%, respectively; χ^2^ *p* = 0.017), however, a post hoc test failed to identify any individual batch with a significant sex difference (*p* ≥ 0.10).

### 2.3. Diet

Before the follow-up study visit, a food diary on four consecutive days including, 1–2 weekend days, was filled in. Participants were instructed to record regular days and non-regular, e.g., holidays/sick days where food intake was atypical. Portion sizes were estimated using household measures (e.g., spoons, cups) or a food picture booklet, and details regarding the foods (e.g., brand and preparation method) were requested. During the study visit, the diary was reviewed for completeness and accuracy by a dietary technician, and missing details were added after discussion where necessary. The food diary data were entered into the Micro-Nutrica^®^ food analysis software (developed at the Research and Development Centre of the Social Insurance Institution, Finland) to calculate food and nutrient intake. This software has been regularly updated throughout the study, and can calculate 66 separate nutrient values from over 4000 foods and dishes.

### 2.4. Other Characteristics

At the follow-up study visit, height, weight and waist circumference were measured and BMI was calculated as weight (kg)/(height (m^2^)). Blood samples were drawn following overnight fasting, and serum samples were separated, aliquoted, and stored at −70 °C. The samples were thawed for the first time for the following analyses. Serum triglycerides, total cholesterol, HDL-cholesterol, and serum glucose were analyzed using an AU400 instrument (Olympus, Hamburg, Germany) and applicable system reagents (Beckman Coulter, Brea, CA, USA). LDL cholesterol concentration was estimated using the Friedewald formula [27]. If triglyceride level was ≥4.5 mmol/L, LDL cholesterol was set to missing. Serum insulin was determined using an ARCHITECT insulin assay (Abbott, Chicago, IL, USA) on an Architect ci8200 analyzer (Abbott, USA), and insulin resistance was estimated using the homeostatic model for assessing insulin resistance (HOMA-IR; fasting insulin × (fasting glucose/22.5)). Sitting blood pressure was measured using an oscillometric device, with an average of three measurements used in the analyses. Data regarding physical activity and smoking habits were collected by questionnaires.

### 2.5. Statistical Analyses

Differences in the dietary measures and cardiometabolic risk markers and between the intervention and control groups were analyzed with R (v. 3.6.2, R Foundation for Statistical Computing, Vienna, Austria; https://www.R-project.org/) using linear models adjusted for sex. For triglycerides, insulin, and HOMA-IR, ln-transformed values were applied. For categorical variables, Pearson’s χ^2^ test was used. To study how well the sample cohort (*n* = 357) represented the entire eligible STRIP 26-year follow-up study cohort (attended study visit; *n* = 546), the same response variables were compared between those with a successfully sequenced fecal sample and those who had participated in the 26-year follow-up study visit and who either had not provided a fecal sample or whose sample had been excluded from the final analyses. Altogether, 35 (9.9%) individuals had self-reported an antibiotic course during the three months prior to sample collection (10.4% in the intervention group and 9.5% in the control group; *p* = 0.42). Adjustment for multiple comparisons was not performed for these analyses.

In order to compare the gut microbiota of the intervention and control groups, R (v. 3.6.2) and the Bioconductor packages *phyloseq* [28], *microbiome* [29], and *vegan* [30] were used. All analyses were performed first for the whole study group and then for males and females separately in order to study sex-specific associations. Gut microbiota alpha diversity, represented by Shannon index and Chao1 richness, was determined using *microbiome*. The Shannon index describes the bacterial diversity in a sample by counting the abundance and evenness of the ASVs present, while Chao1 estimates the bacterial richness in a sample based on the abundance of unique ASVs.

Microbiome beta diversity, which describes the dissimilarities in ecosystem level community composition between samples, was analyzed with Permutational Analysis of Variation (PERMANOVA) using the adonis function in *vegan* and Bray–Curtis dissimilarity. The beta diversity analyses were performed for ASV-level phyloseq objects, which were transformed into compositionals; 99 permutations were used. The *p*-values were adjusted for multiple testing with the Benjamini and Yekutieli procedure. For visualization, Principal Coordinates Analysis (PCoA) plots were generated using *microbiome*. Analyses regarding the taxonomy of the microbiota were performed using *DESeq2*, which uses shrinkage estimation for dispersions and fold changes to perform quantitative analysis of differential expression. In order to reduce multiple comparisons, rare taxa were excluded prior to *DESeq2* analysis by filtering out ASVs with a relative abundance <0.1% in >98% of the samples, resulting in a phyloseq object including 423 unique ASVs corresponding to nine different bacterial phyla and 77 bacterial genera. We analyzed differential abundances on the phylum, family, genus, and species level. In *DESeq2* analyses, *p*-values were adjusted for multiple comparison using the Benjamini–Hochberg procedure, and we considered adjusted *p*-values at a level of 0.05 to be statistically significant [31].

## 3. Results

The study cohort consisted of 357 26-year-old individuals, of whom 154 (43.1%) were males and 174 (48.7%) belonged to the intervention group. Characteristics of the dietary measures and cardiometabolic risk markers of the participants by belonging to the intervention or control group are presented in Table 1. Individuals in the intervention group had a lower intake of SAFA, and they tended to have a higher ratio of PUFA + monounsaturated fat (MUFA) to SAFA and to consume more vegetables, fruit, and berries daily compared to the control group (Table 1). No differences in BMI, waist circumference, physical activity, or prevalence of regular smoking were observed between the groups. In sex-specific analyses, the effect of the intervention on SAFA was evident in females, while males in the intervention group had higher intake of fiber (g/MJ) and of vegetables, fruit, and berries compared with control group males (Table S1). The participants with successful gut microbiota analyses (*n* = 357) consumed more vegetables, fruit, and berries and tended to have higher total daily fiber intake compared to individuals who had attended the 26-year follow-up study clinic visit and either did not provide a fecal sample or provided a sample that could not be successfully sequenced (*n* = 189; Table S2). Other dietary and anthropometric characteristics were similar between the groups.

### 3.1. 20-Year Dietary Counselling: Microbiota Alpha and Beta Diversity Six Years Post-Intervention

No statistically significant differences in microbiota alpha diversity, i.e., Shannon index or Chao1 richness, were observed between the intervention and control group participants (Kruskal–Wallis *p* = 0.081 and *p* = 0.27, respectively; see Table 2). Furthermore, no differences were observed between males and females in terms of Shannon index (median 3.48 and 3.66, respectively; Kruskal–Wallis *p* = 0.097) or Chao1 (median 239 and 250, respectively; Kruskal–Wallis *p* = 0.30). In the same line, no differences were observed between the study groups when males and females were analyzed separately (Table 2).

**Figure 2:**
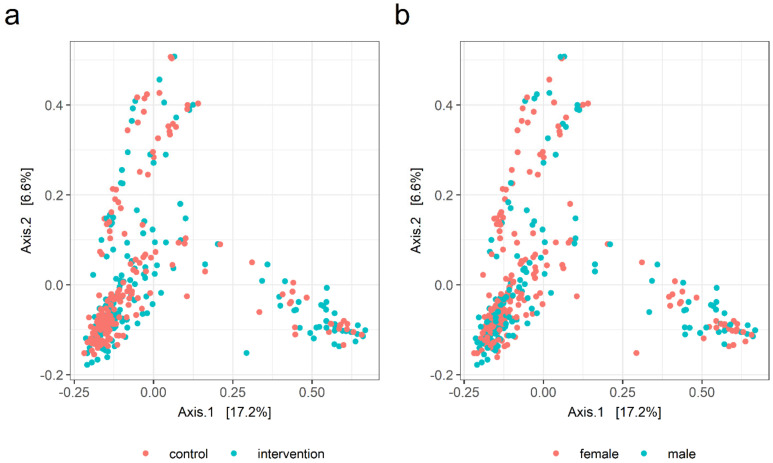
PCoA ordination plots for gut microbiota beta diversity: (**a**) in the dietary counselling intervention and control groups (PERMANOVA R^2^ = 0.004, *p* = 0.09) and (**b**) for females and males (R^2^ = 0.005, *p* = 0.01).

With respect to microbiota beta diversity, no apparent differences were visible in the PCoA ordination between the intervention and control groups when both males and females were included in the analyses (Figure 2a). PERMANOVA substantiated this visual observation, indicating that the study group did not explain a significant proportion of variation in the ecosystem-level fecal microbiota profiles (PERMANOVA R^2^ = 0.004; *p* = 0.09). Although no evident difference between males and females could be visually observed in the PCoA plot (Figure 2b), microbiota beta diversity appeared to differ by sex (R^2^ = 0.005; *p* = 0.01). When only male participants were included in the PERMANOVA analysis, a marginally significant difference in beta diversity was observed between the intervention and the control groups (R^2^ = 0.010; *p* = 0.05). In females, on the other hand, no difference in the fecal microbiota beta diversity was observed between the study groups (R^2^ = 0.004; *p* = 0.66).

### 3.2. Microbial Taxa in the Intervention and Control Groups

After removal of extremely rare microbial taxa, nine different bacterial phyla and 77 bacterial genera were detected in the study population. *Bacteroidetes* (mean relative abundance 52.0%) and *Firmicutes* (40.6%) were the most abundant phyla, followed by *Proteobacteria* (4.0%) and *Actinobacteria* (2.7%). Of the observed genera, *Bacteroides* (mean relative abundance 18.4%), *Prevotella* (18.4%), and *Faecalibacterium* (7.3%) were the most abundant. The mean relative abundances of all observed phyla and genera are presented in Table S3.

**Figure 3:**
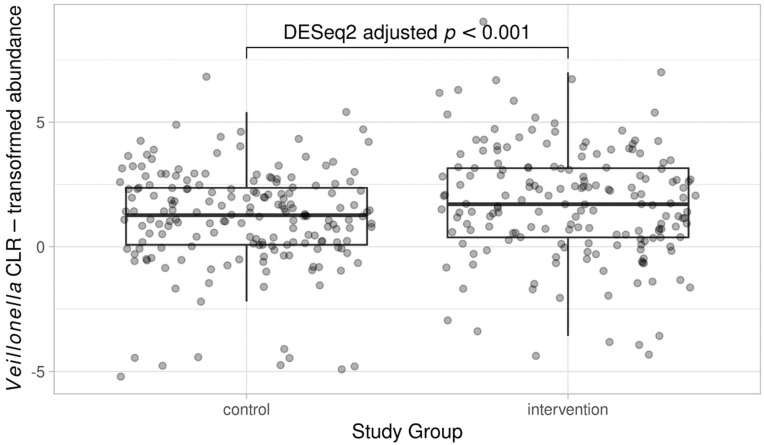
Abundance of genus *Veillonella* in the dietary counselling intervention and control groups. For illustration, CLR-transformed count data is used.

In *DESeq2*, no phylum-level differences were observed between the study groups when males and females were analyzed together (Figure S1). However, the abundance of one bacterial family, *Veillonellaceae*, was elevated in the intervention group compared to the control group (log_2_ fold-change 0.75, adjusted *p* = 0.007), and genus *Veillonella* was consistently and significantly more abundant in the intervention group (log_2_ fold-change 1.58, adjusted *p* < 0.001; Figure 3). No other family or genus-level bacterial signatures with significant association with the study group were observed. Retrospective species-level inspection suggested that the observed differences in the abundance of genus *Veillonella* may have originated from two distinct *Veillonella* species, namely, *V. dispar* and *V. rogosae*, the abundance of which varied between the study groups (log_2_ fold-change 1.31 and adjusted *p* < 0.001 vs. log_2_ fold-change 1.31 and adjusted *p* = 0.003, respectively). In addition, the abundance of the species *Flavonifractor plautii* appeared to differ between the study groups (log_2_ fold-change 0.82, adjusted *p* = 0.028).

When only males were included in the DESeq2 analysis, the phylum *Bacteroidetes* tended to be slightly more abundant in the intervention group (log_2_ fold-change 0.70, adjusted *p* = 0.061). In females, no phylum-level differences were observed between the study groups. Furthermore, at the bacterial genus level the previously observed difference in the abundance of *Veillonella* persisted when males and females were analyzed separately. In females, *Veillonella* was the only genus-level finding between the intervention and control group participants (log_2_ fold-change 1.47, adjusted *p* < 0.001), while in males, in addition to *Veillonella* (log_2_ fold-change 1.67, adjusted *p* < 0.001), the genus *Intestinibacter* was found to be more abundant in the intervention group as compared to the control group (log_2_ fold-change 1.70, adjusted *p* = 0.020). However, the abundance of *Intestinibacter* was extremely low in general (max relative abundance 0.8%, mean relative abundance 0.0%).

## 4. Discussion

This study shows that a repeated 20-year infancy-onset dietary counselling program induced bacterial signatures in the gut microbiota profile as observed six years after cessation of the intervention. The intervention group participants possessed elevated levels of family *Veillonellaceae* and genus *Veillonella* compared to controls. Furthermore, the males in the intervention group tended to have higher beta diversity and phylum *Bacteroidetes* abundance compared to the control group males. As studies linking long-term dietary interventions with gut microbiota are scarce and similar intervention settings extending two decades after infancy are non-existent, these results add novel knowledge about the long-term effects of a dietary counselling intervention aimed at inducing a heart-healthy diet.

In the STRIP study, evident beneficial effects of repeated dietary counselling on diet and cardiometabolic health have been observed during the 20-year intervention period [23,32,33], and the intervention’s effects were largely maintained six years post-intervention [24]. For those who comprised the study cohort applied here (*n* = 357, 65% of the eligible sample), we observed that the individuals in the intervention group had a lower intake of SAFA and a slightly higher PUFA + MUFA to SAFA ratio (>2:1) compared to the control group, and consumed more vegetables, fruit, and berries.

Concerning the present study of gut microbial composition, the most evident gut bacterial signature observed was the higher abundance of the genus *Veillonella* in the intervention group participants compared to the controls. To the best of our knowledge, similar holistic dietary interventions aiming to improve dietary fat quality and increase fiber intake, such as that characteristic of the Mediterranean diet, have not reported increased abundance of *Veillonella* [34]. The members of the *Veillonella* genus are Gram-negative, non-spore-forming, non-motile, and strictly anaerobic cocci that belong to both the commensal gut and oral microbiota [35]. While *Veillonella* form biofilms and can be associated with human infections, they are generally considered to be of low virulence [35]. *Veillonella* are lactate-degrading bacteria that ferment lactates to short-chain fatty acids, mainly propionate [36]. Interestingly, a recent study has proposed that bacteria within this particular genus have physical performance-enhancing properties linked to enhanced lactate metabolism in both humans and rodents [36]. We found, however, that there was no difference in physical activity level between the study groups, suggesting that the higher abundance of *Veillonella* in the intervention group is more likely associated with diet than with exercise practices. No single evident diet-related explanation for the higher abundance of *Veillonella* could be determined, although it could be related to, for example, a higher abundance of lactic acid-producing bacteria in the gut. The abundance of lactate-producing *Lactobacillus* has previously been reported to increase as a consequence of prebiotic fiber intake [37], and *Veillonella* are known to utilize lactate as their main carbon source [35]. In the present study, no differences in the abundance of any lactic acid bacteria representing genera were observed between the intervention and control groups, which may be explained by the fact that lactic acid bacteria reside in several separate bacterial taxonomic families, and thus deep shotgun sequencing would be needed in order to provide more precise analysis of their abundance.

Interestingly, our results revealed more distinct variation between the intervention and control groups’ gut microbiota profiles in males. Specifically, we noted that the ecosystem-level gut microbiota profiles varied between the intervention and control group in males, and did not differ in females. This observation may be related to higher fiber intake in the intervention group males as compared to controls, which was not observed for females. Because dietary fiber serves as the substance for microbial fermentation, its intake affects the composition of gut microbiota and can, for instance, increase the abundance of *Bacteroides* species [6,38,39]. The higher fiber intake among the intervention compared to control group males is likely an indication of their higher consumption of vegetables, fruit, and berries, which evidently contain various other components in addition to fiber, for instance, polyphenols, which have been speculated to modulate gut microbiota [40]. Thus, the differences in fiber intake may underlie the observed differences in the gut microbiota profiles and the modestly higher *Bacteroides* abundance in intervention group males, which is in line with previous observational and dietary intervention studies [34,41]. Collectively, even though no striking differences in the taxonomic microbiota profiles were observed between the study groups, it is possible that functional gut microbiota analysis such as deep metagenomic sequencing or metabolomics would have revealed differences in the metabolic pathways of the gut microbes, specifically, those related to carbohydrate degradation [42,43].

Dietary and lifestyle interventions have been widely acknowledged to reduce cardiometabolic risk factors in both children and adults [44,45]. Concomitantly, individuals with CVDs have been reported to harbor altered gut microbiota composition compared to healthy controls [46,47], and atherosclerotic plaques have been reported to contain bacterial DNA that possibly originates from the gut [48]. Perturbations of the gut microbiome are generally associated with intestinal inflammation and reduced gut barrier integrity, which enables excess leakage of bacterial structural components and microbial metabolites from the intestines [49]. Consequently, this state of ‘dysbiosis’ has been proposed to promote the development of CVDs [47,50]. One common theory linking gut microbiota and CVDs is that high circulating levels of microbial metabolites, such as trimethylamine N-oxide (TMAO) and its precursors, predispose individuals to atherosclerosis by, e.g., affecting the cholesterol metabolism [51]. The underlying mechanisms, however, remain largely uncertain.

In addition, it remains undefined whether and how certain food constituents, such as different types of dietary fats, can promote the growth and function of so-called beneficial gut microbes or cause a predisposition to adverse microbiota changes [6,11,12]. Furthermore, no other dietary interventions similar to STRIP aiming to holistically promote a heart-healthy diet from infancy have been conducted. In prior intervention and observational studies, the Mediterranean diet, characterized by a more favorable dietary fat quality and fiber intake compared to a traditional Western diet, has been suggested to increase the abundances of *Prevotella*, *Bacteroides* [34,52,53], and *Lachnospiraceae*, among other genera [54]. However, these observations have not been systematically evident in all studies [41]. In our study, a modest increase in *Bacteroides* abundance in males was detected, which may be linked to the relatively small difference in fiber intake between the groups. Moreover, even though *Bifidobacterium* and *Lactobacillus* have previously been linked to the intake of unsaturated fats [15,17], we did not observe any differences in the abundances of these taxa despite the modestly improved quality of dietary fats in the intervention group (i.e., higher intake of unsaturated compared to saturated fats). In order to clarify the underlying mechanisms between diet and gut microbiota, long-term dietary interventions coupled with temporal gut microbiota sampling are required. The results of this study suggest that decreased intake of SAFA, increased intake of fiber, and an altogether slightly improved dietary profile brings about small differences in the fecal microbiota composition. Our results corroborate that the intervention based on dietary recommendations was not detrimental for the microbial inhabitants of the gut.

Diet switches represent an effective natural way of modifying both the gut microbiota and the metabolic health of an individual, as these further modify the composition and metabolic functions of the gut microbiota, especially in the large intestine [6,14]. Modulation of gut microbiota composition by diet thus represents a promising non-invasive therapeutic target for, e.g., metabolic diseases. Dietary interventions have, for example, shown promise in reducing T2D and CVD risk [55,56,57], and these findings may, at least in part, be related to the modulation of gut microbiota. However, these studies lack data on gut microbiota, and although short-term dietary interventions spanning 6 to 12 months have the capability of transiently altering the composition of the gut microbiota [9,58], it seems that long-term dietary changes are required for more permanent shifts in the structure of the gut microbiota [8,9,10]. Interestingly, it may be the case that the intervention effects are not sustained, as, for instance, 12-month low-carbohydrate or low-fat dietary interventions resulted in an initial change in the gut microbiota composition that returned to the baseline state during the intervention period [59]. The resilience of adult gut microbiota might be circumvented with interventions targeting nutrition in early life, when the gut microbiome composition is developing [60]. Ideally, interventions targeted to the prevention of CVDs would thus span several years, begin early in life, and simultaneously collect gut microbial data in order to provide evidence as to whether dietary modification of the gut microbial ecosystem provides a mechanistic link between diet and cardiovascular risk factors and phenotypes.

The main limitation of this study is that only a single fecal sample from each participant was collected. As dietary changes can modify the gut microbiota composition relatively rapidly [6,14], a single sample may not capture the ecosystem-level differences between individuals accurately [61]. Furthermore, as no fecal samples were collected during the intervention period, it remains uncertain whether the differences in microbiota between the intervention and the control groups would have been more prominent during the intervention period. Another limitation is that the fecal microbiota composition analysis was performed using 16S rRNA gene sequencing, a method which describes only the taxonomic abundances of the bacteria, and cannot reliably be used for estimating their metabolic capability or function [62]. It may be that the diet had a greater influence on the metabolic activity than on the taxonomic composition of the gut microbiota. Moreover, 16S rRNA gene sequencing does not provide reliable species-level identification. However, regarding the 16S rRNA gene sequencing data, DADA2 was used to infer amplicon sequence variants, which increases the reliability of the obtained results at lower taxonomic levels [26]. In addition, this study has a well-characterized and credible sample population (*n* = 357), especially as relates to the detailed assessment of diet. As another limitation, we acknowledge that the individuals who provided a fecal sample that was successfully sequenced and those who either did not provide a fecal sample or whose sample was excluded from the final analyses were not similar related in terms of all assessed characteristics. Furthermore, the assessment of diet was based on self-reporting, and we did not control for disease or lifestyle characteristics. The key strength of this study is its unique longitudinal intervention setting, particularly in light of the fact that data on the effects of long-term dietary interventions on gut microbiota remain scarce.

## 5. Conclusions

This study shows that an infancy-onset repeated 20-year dietary counselling program may induce subtle changes in the gut microbiota six years post-intervention. We observed that the associations in the study group were more prominent among males, possibly reflecting differences in dietary fiber intake. The observed differences between the intervention and control groups reflect favorable gut microbial changes, thus further confirming the safety of the provided dietary counselling. Speculatively, the benefits of the intervention may in part be related to the modulation of gut microbiota.

## Figures and Tables

**Table 1:** Cardiometabolic risk markers and dietary measures of the participants six years post-intervention by intervention or control group (*n* = 357; age 26 years). The presented values are mean (SD), except for triglycerides, insulin, and HOMA-IR, for which median (IQR) are shown, and for smoking, where prevalence of daily smokers is reported (%). *p*-values are adjusted for sex.

	Intervention (*n* = 174)	Control (*n* = 183)	*p*-Value
Sex, males (%)/females (%)	81 (46.6)/93 (53.4)	73 (39.9)/110 (60.1)	0.24 ^#^
Body mass index, kg/m^2^	24.3 (4.0)	24.3 (4.4)	0.83
Waist circumference, cm	80.5 (11.0)	80.4 (11.9)	0.57
Systolic blood pressure, mmHg	122.2 (11.0)	119.5 (11.5)	0.074
Diastolic blood pressure, mmHg	72.0 (7.8)	71.8 (7.6)	0.97
**Dietary intakes**	***n* = 155**	***n* = 171**	
Energy, kcal/day	2010 (581)	2044 (576)	0.19
Protein, E%	19.8 (5.3)	19.3 (4.9)	0.54
Carbohydrates, E%	41.6 (8.5)	40.4 (7.4)	0.11
Sucrose, E%	5.7 (2.9)	6.3 (3.6)	0.18
Fat, E%	36.9 (7.4)	38.1 (6.9)	0.11
SAFA, E%	12.9 (3.33)	14.1 (3.6)	0.003
MUFA, E%	13.2 (4.1)	13.1 (3.6)	0.99
PUFA, E%	6.7 (2.2)	6.6 (2.1)	0.72
(P + M)/S	1.61 (0.51)	1.49 (0.58)	0.051
P/S	0.55 (0.25)	0.51 (0.25)	0.084
Cholesterol, mg/day	300 (179)	325 (200)	0.089
Fiber, g/day	20.1 (8.1)	19.6 (8.1)	0.53
Fiber, g/MJ	2.44 (0.81)	2.35 (0.89)	0.16
Fiber rich grains, g/day	74.8 (41.4)	68.2 (44.8)	0.27
Vegetables, fruit, and berries, g/day	376 (223)	337 (209)	0.052
Sodium, mg/day	2938 (934)	2996 (1050)	0.20
**Physical activity**	***n* = 168**	***n* = 174**	
MET h/wk	25.0 (17.7)	24.1 (20.4)	0.76
**Smoking**	***n* = 169**	***n* = 176**	
Daily smoking, %	6.5	8.0	0.61
**Serum biomarkers**	***n* = 173**	***n* = 183**	
Total cholesterol, mmol/L	4.48 (0.83)	4.63 (0.97)	0.14
HDL cholesterol, mmol/L	1.30 (0.33)	1.38 (0.35)	0.081
LDL cholesterol, mmol/L	2.70 (0.71)	2.82 (0.78)	0.13
Triglycerides, mmol/L	0.90 [0.60]	0.80 [0.50]	0.14
Insulin, mU/L	6.5 [3.9]	7.1 [4.0]	0.090
Glucose, mmol/L	5.0 (0.49)	5.0 (0.69)	0.54
HOMA-IR	1.42 [0.98]	1.59 [0.97]	0.080

SAFA, saturated fatty acids; MUFA, monounsaturated fatty acids; PUFA, polyunsaturated fatty acids; E%, percentage of energy intake; (P + M)/S, polyunsaturated and monounsaturated fat to saturated fat ratio; P/S, polyunsaturated fat to saturated fat ratio; HOMA-IR, homeostatic model assessment of insulin resistance. ^#^ Pearson’s χ^2^ test.

**Table 2:** Gut microbiota alpha diversity; values are median (range).

Study Cohort (*n* = 357)	Intervention (*n* = 174)	Control (*n* = 183)	*p*-Value ^#^
Read count	157 k (11.9 k–752 k)	165 k (19.7 k–839 k)	0.092
Shannon index	3.5 (0.5–4.5)	3.7 (0.4–4.5)	0.081
Chao 1 richness	245 (76–454)	247 (82–547)	0.27
**Males (*n* = 154)**	**Intervention (*n* = 81)**	**Control (*n* = 73)**	***p*-Value ^#^**
Read count	160 k (11.9 k–255 k)	171 k (19.7 k–839 k)	0.11
Shannon index	3.4 (0.7–4.5)	3.6 (0.4–4.4)	0.20
Chao 1 richness	238 (76–431)	247 (82–423)	0.23
**Females (*n* = 203)**	**Intervention (*n* = 93)**	**Control (*n* = 110)**	***p*-Value ^#^**
Read count	156 k (25.0 k–752 k)	163 k (20.1 k–705 k)	0.33
Shannon index	3.6 (0.5–4.5)	3.7 (0.6–4.5)	0.29
Chao 1 richness	250 (90–454)	253 (91–547)	0.72

^#^ Kruskal–Wallis test.

## Data Availability

The dataset supporting the conclusions of this article were obtained from the STRIP study. The STRIP dataset comprises health-related participant data, and its use is therefore restricted under the regulations on professional secrecy (Act on the Openness of Government Activities, 612/1999) and on sensitive personal data (Personal Data Act, 523/1999, implementing the EU data protection directive 95/46/EC). Due to these legal restrictions, the data from this study cannot be stored in public repositories or otherwise made publicly available. However, data access may be permitted on a case-by-case basis upon request only. Data sharing outside the group is carried out in collaboration with the STRIP group and requires a data-sharing agreement. Investigators can submit an expression of interest to the chairman of the STRIP steering group (Prof Olli Raitakari, University of Turku, Turku, Finland).

## Supplementary Materials

The following supporting information can be downloaded at: https://www.mdpi.com/article/10.3390/nu14132667/s1, Table S1: Cardiometabolic risk markers and dietary measures of the female and male participants six years post-intervention by intervention and control group (age 26 years). Values are presented as mean (SD), except for triglycerides, insulin, and HOMA-IR, for which median (IQR) values are shown; Table S2: Comparison of STRIP 26-year follow-up study participants who provided a fecal sample that was successfully sequenced (*n* = 357) and those who either did not provide a fecal sample or whose sample was excluded from the final analyses (*n* = 189). Values are presented as mean (SD), except for triglycerides, insulin, and HOMA-IR, for which median (IQR) values are shown. *p*-values are adjusted for sex; Table S3: Relative abundance of gut microbes on phylum and genus level in the cohort; Figure S1: Relative abundance of bacterial phyla according to the dietary counselling intervention and control groups.
