# Supplementary material for: An Infancy-Onset 20-Year Dietary Counselling Intervention and Gut Microbiota Composition in Adulthood
Source: Nutrients. 2022 Jun 27;14(13):2667. doi: 10.3390/nu14132667 (PMC9268486; doi:10.3390/nu14132667)
Supplement: Supplementary file 1 [file nutrients-14-02667-s001.zip › Figure S1.pdf]

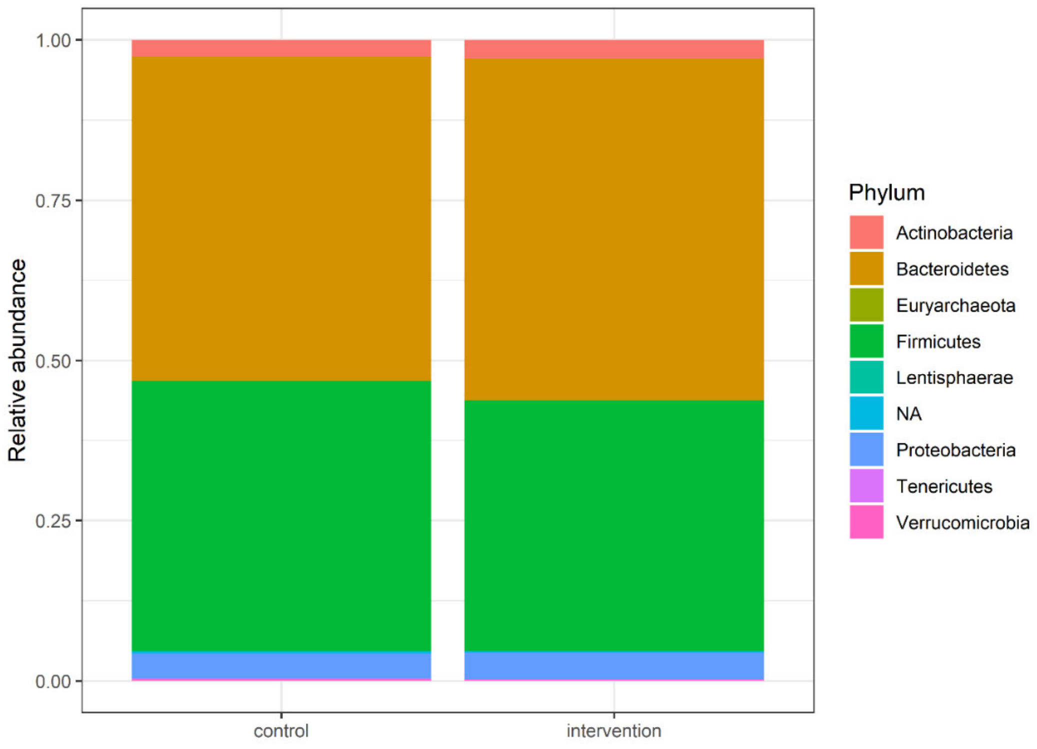

Figure S1. Relative abundances of bacterial phyla according to the dietary counselling intervention and control groups.
