# Supplementary material for: An Infancy-Onset 20-Year Dietary Counselling Intervention and Gut Microbiota Composition in Adulthood
Source: Nutrients. 2022 Jun 27;14(13):2667. doi: 10.3390/nu14132667 (PMC9268486; doi:10.3390/nu14132667)
Supplement: Supplementary file 1 [file nutrients-14-02667-s001.zip › Table S1.pdf]

**Table S1.** Cardiometabolic risk markers and dietary measures of the female and male participants six years post-intervention by belonging to the intervention or control group (age 26 years). The presented values are mean (SD), except for triglycerides, insulin, and HOMA-IR, for which median [IQR] are shown.

| | Females | | | Males | | |
|---|---|---|---|---|---|---|
| | **Intervention** | **Control** | **p-value** | **Intervention** | **Control** | **p-value** |
| | n=93 | n=110 | | n=81 | n=73 | |
| **Body mass index, kg/m²** | 23.3 (3.6) | 23.7 (4.7) | 0.56 | 25.4 (4.3) | 25.2 (3.9) | 0.72 |
| **Waist circumference, cm** | 75.0 (8.2) | 75.8 (11.2) | 0.55 | 86.9 (10.3) | 87.2 (9.5) | 0.86 |
| **Systolic blood pressure, mmHg** | 116.4 (7.5) | 115.0 (10.1) | 0.25 | 128.7 (10.8) | 126.4 (10.0) | 0.17 |
| **Diastolic blood pressure, mmHg** | 70.8 (6.2) | 70.5 (7.5) | 0.76 | 73.4 (9.1) | 73.8 (7.2) | 0.73 |
| **Dietary intakes** | n=84 | n=106 | | n=71 | n=65 | |
| **Energy, kcal/day** | 1803 (414) | 1815 (448) | 0.85 | 2256 (652) | 2419 (569) | 0.12 |
| **Protein, E%** | 18.8 (5.3) | 18.6 (4.2) | 0.76 | 21.0 (5.1) | 20.4 (5.8) | 0.57 |
| **Carbohydrates, E%** | 43.1 (8.2) | 41.7 (6.9) | 0.20 | 39.8 (8.5) | 38.4 (7.9) | 0.35 |
| **Sucrose, E%** | 6.4 (2.6) | 6.9 (3.7) | 0.32 | 4.9 (3.1) | 5.4 (3.3) | 0.36 |
| **Fat, E%** | 36.5 (7.0) | 38.3 (6.2) | 0.058 | 37.3 (7.8) | 37.8 (7.9) | 0.71 |
| **SAFA, E%** | 12.6 (3.4) | 14.0 (3.6) | 0.0070 | 13.3 (3.2) | 14.1 (3.7) | 0.18 |
| **MUFA, E%** | 13.1 (4.2) | 13.1 (3.6) | 0.94 | 13.3 (4.0) | 13.2 (3.6) | 0.92 |
| **PUFA, E%** | 6.6 (2.0) | 6.7 (2.2) | 0.86 | 6.8 (2.3) | 6.5 (1.9) | 0.44 |
| **(P+M)/S** | 1.65 (0.56) | 1.52 (0.69) | 0.18 | 1.56 (0.43) | 1.44 (0.36) | 0.10 |
| **P/S** | 0.57 (0.28) | 0.52 (0.28) | 0.26 | 0.54 (0.22) | 0.49 (0.18) | 0.14 |
| **Cholesterol, mg/day** | 253 (134) | 270 (145) | 0.40 | 357 (207) | 415 (242) | 0.14 |
| **Fiber, g/day** | 19.7 (7.6) | 19.9 (8.7) | 0.88 | 20.6 (8.6) | 19.0 (6.9) | 0.23 |
| **Fiber, g/MJ** | 2.62 (0.83) | 2.63 (0.91) | 0.95 | 2.22 (0.72) | 1.91 (0.64) | 0.0084 |
| **Fiber rich grains, g/day** | 65.6 (38.0) | 63.3 (36.5) | 0.67 | 85.6 (42.8) | 76.3 (55.0) | 0.27 |
| **Vegetables, fruit, and berries, g/day** | 398 (217) | 388 (222) | 0.76 | 350 (230) | 255 (153) | 0.0055 |
| **Sodium, mg/day** | 2552 (644) | 2630 (777) | 0.46 | 3396 (1017) | 3593 (1163) | 0.30 |
| **Physical activity** | n=92 | n=105 | | n=76 | n=69 | |
| **MET h/wk** | 23.2 (15.5) | 22.2 (17.7) | 0.67 | 27.2 (20.0) | 27.1 (23.8) | 0.98 |
| **Smoking** | n=92 | n=106 | | n=77 | n=70 | |
| **Daily smoking, %** | 5.4 | 5.7 | 0.94 | 7.8 | 11.4 | 0.45 |
| **Serum biomarkers** | n=92 | n=110 | | n=81 | n=73 | |
| **Total cholesterol, mmol/L** | 4.62 (0.78) | 4.68 (0.99) | 0.65 | 4.32 (0.86) | 4.57 (0.93) | 0.084 |
| **HDL cholesterol, mmol/L** | 1.46 (0.31) | 1.47 (0.36) | 0.85 | 1.12 (0.26) | 1.24 (0.28) | 0.0059 |
| **LDL cholesterol, mmol/L** | 2.73 (0.65) | 2.78 (0.77) | 0.59 | 2.67 (0.78) | 2.88 (0.80) | 0.11 |
| **Triglycerides, mmol/L** | 0.90 [0.60] | 0.80[0.60] | 0.71 | 1.00 [0.60] | 0.80 [0.40] | 0.076 |
| **Insulin, mU/L** | 6.3 [3.1] | 7.2 [3.7] | 0.095 | 6.5 [4.9] | 6.9 [4.6] | 0.42 |
| **Glucose, mmol/L** | 4.91 (0.41) | 4.99 (0.48) | 0.24 | 5.14 (0.89) | 5.13 (0.50) | 0.94 |
| **HOMA-IR** | 1.42 [0.79] | 1.62 [0.90] | 0.082 | 1.43 [1.25] | 1.58 [1.03] | 0.43 |

SAFA, saturated fatty acids; MUFA, monounsaturated fatty acids; PUFA, polyunsaturated fatty acids; E%, percentage of energy intake; (P+M)/S, polyunsaturated and monounsaturated fat to saturated fat ratio; P/S, polyunsaturated fat to saturated fat ratio; HOMA-IR, homeostatic model assessment of insulin resistance.
