# Supplementary material for: An Infancy-Onset 20-Year Dietary Counselling Intervention and Gut Microbiota Composition in Adulthood
Source: Nutrients. 2022 Jun 27;14(13):2667. doi: 10.3390/nu14132667 (PMC9268486; doi:10.3390/nu14132667)
Supplement: Supplementary file 1 [file nutrients-14-02667-s001.zip › Table S2.pdf]

Table S2. Comparison of those STRIP 26-year follow-up study participants who provided a fecal sample that was successfully sequenced (n=357) and those who either did not provide a fecal sample or whose sample was excluded from the final analyses (n=189). The presented values are mean (SD), except for triglycerides, insulin, and HOMA-IR, for which median [IQR] are shown. P-values are adjusted for sex.

	Fecal sample sequenced (n = 357)	Fecal sample not sequenced (n = 189)	p-value
**BMI, kg/m^2**	24.3 (4.24)	24.8 (4.63)	0.20
**Waist circumference, cm**	80.4 (11.5)	82.2 (11.1)	0.12
**Systolic blood pressure, mmHg**	120.8 (11.3)	120.9 (10.0)	0.76
**Diastolic blood pressure, mmHg**	71.9 (7.67)	72.0 (6.85)	0.92
	**n = 342**	**n = 167**	
**Physical activity, MET h/wk**	24.6 (19.1)	24.3 (18.5)	0.88
	**n = 356**	**n = 188**	
**Total cholesterol, mmol/L**	4.56 (0.90)	4.69 (0.88)	0.10
**HDL cholesterol, mmol/L**	1.34 (0.34)	1.33 (0.29)	0.68
**LDL cholesterol, mmol/L**	2.76 (0.75)	2.87 (0.76)	0.11
**Triglycerides, mmol/L**	0.9 [0.5]	1.0 [0.6]	0.081
**Insulin, mU/L**	6.7 [3.9]	7.1 [4.0]	0.40
**Glucose, mmol/L**	5.0 (0.59)	5.1 (1.09)	0.26
**HOMA-IR**	1.5 [1.0]	1.6 [1.0]	0.33
	**n = 326**	**n = 146**	
**Total energy intake, kcal/day**	2028 (578)	1913 (573)	0.093
**Protein, E%**	19.5 (5.11)	19.0 (5.39)	0.40
**Carbohydrates, E%**	41.0 (7.96)	41.6 (7.97)	0.53
**Sucrose, E%**	6.0 (3.3)	6.0 (3.3)	0.74
**Fat, E%**	37.5 (7.14)	36.8 (6.84)	0.31
**SAFA, E%**	13.5 (3.51)	13.3 (3.48)	0.56
**MUFA, E%**	13.1 (3.8)	12.7 (3.4)	0.25
**PUFA, E%**	6.6 (2.2)	6.6 (2.2)	1.00
**(P+M)/S**	1.55 (0.55)	1.54 (0.51)	0.84
**P/S**	0.53 (0.25)	0.54 (0.26)	0.68
**Dietary cholesterol, mg/day**	313 (190)	290 (175)	0.35
**Fiber intake, g/day**	19.8 (8.1)	18.2 (8.8)	0.052
**Fiber intake, g/MJ**	2.39 (0.85)	2.32 (0.99)	0.26
**Fiber rich grains, g/day**	71.3 (43.2)	70.5 (44.0)	1.00
**Vegetables, fruit, and berries, g/day**	355 (216)	295 (226)	0.003
**Sodium, mg/day**	2968 (995)	2769 (927)	0.083

SAFA, saturated fatty acids; MUFA, monounsaturated fatty acids; PUFA, polyunsaturated fatty acids; E%, percentage of energy intake; (P+M)/S, polyunsaturated and monounsaturated fat to saturated fat ratio; HOMA-IR, homeostatic model assessment of insulin resistance.
