# Supplementary material for: An Infancy-Onset 20-Year Dietary Counselling Intervention and Gut Microbiota Composition in Adulthood
Source: Nutrients. 2022 Jun 27;14(13):2667. doi: 10.3390/nu14132667 (PMC9268486; doi:10.3390/nu14132667)
Supplement: Supplementary file 1 [file nutrients-14-02667-s001.zip › Table S3.pdf]

Table S3. Relative abundances of gut microbes on phylum and genus level in the cohort.

	Phylum	Mean (%)	Median (%)	Min (%)	Max (%)
1	*Bacteroidetes*	52	46.2	0.4	97.5
2	*Firmicutes*	40.6	44.2	1.8	95.7
3	*Proteobacteria*	4	3.4	0	24
4	*Actinobacteria*	2.7	1.3	0	51.6
5	NA	0.3	0	0	10
6	*Verrucomicrobia*	0.2	0	0	4.3
7	*Tenericutes*	0.1	0	0	9.1
8	*Euryarchaeota*	0	0	0	2.4
9	*Lentisphaerae*	0	0	0	0.6
	**Genus**	**Mean (%)**	**Median (%)**	**Min (%)**	**Max (%)**
1	*Bacteroides*	18.4	17.3	0	59.1
2	*Prevotella*	18.4	0.2	0	96.6
3	*Faecalibacterium*	7.3	6.5	0	27.8
4	*Barnesiella*	5.6	0.7	0	92.1
5	*Roseburia*	5.3	4	0	30.6
6	*Paraprevotella*	4.1	0	0	61.6
7	*Clostridium* XlVa	3.5	2.8	0.1	27.9
8	*Lachnospiracea incertae sedis*	3.1	2.4	0	24.6
9	*Phascolarctobacterium*	2.8	0	0	45
10	*Alistipes*	2.5	1.9	0	34
11	*Bifidobacterium*	2.2	0.8	0	51.6
12	*Ruminococcus*	2.2	1.2	0	17.8
13	*Bacteria_Firmicutes_Clostridia_Clostridiales_Lachnospiraceae*_NA	1.8	1.4	0	14.6
14	*Sutterella*	1.7	0.9	0	14.4
15	*Bacteria_Firmicutes_Clostridia_Clostridiales_Ruminococcaceae*_NA	1.5	1	0	15
16	*Parabacteroides*	1.4	1	0	17.8
17	*Blautia*	1.2	0.9	0	12.2
18	*Fusicatenibacter*	1.1	0.8	0	7.2
19	*Oscillibacter*	1.1	0.8	0	19.6
20	*Odoribacter*	1	0.7	0	5.6
21	*Coprococcus*	1	0.5	0	6.8
22	*Bacteria_Firmicutes_Clostridia_Clostridiales*_NA_NA	0.9	0.5	0	9.2
23	*Clostridium IV*	0.9	0.4	0	12.5
24	*Gemmiger*	0.9	0.4	0	13.8
25	*Parasutterella*	0.8	0.1	0	11.2
26	*Ruminococcus2*	0.6	0.3	0	5.3
27	*Bacteria_Firmicutes*_NA_NA_NA_NA	0.6	0.1	0	5.8
28	*Dialister*	0.6	0.3	0	5.2
29	*Eubacterium*	0.5	0.4	0	5.3

**30**	*Collinsella*	0.4	0.2	0	5.9
**31**	*Veillonella*	0.4	0.1	0	12.9
**32**	*Escherichia/Shigella*	0.4	0	0	23.7
**33**	*Butyricimonas*	0.3	0	0	2.6
**34**	*Coprobacter*	0.3	0	0	3.9
**35**	*Dorea*	0.3	0.2	0	3.6
**36**	*Flavonifractor*	0.3	0.2	0	6.5
**37**	*Sporobacter*	0.3	0.1	0	4.7
**38**	*Bacteria*_NA_NA_NA_NA_NA	0.3	0	0	10
**39**	*Streptococcus*	0.2	0.1	0	2.8
**40**	*Clostridium sensu stricto*	0.2	0	0	5.8
**41**	*Anaerostipes*	0.2	0.1	0	2.5
**42**	*Clostridium* XlVb	0.2	0.1	0	2.5
**43**	*Clostridium* III	0.2	0	0	5.8
**44**	*Acidaminococcus*	0.2	0	0	16.8
**45**	*Succiniclasticum*	0.2	0	0	19
**46**	*Bacteria_Proteobacteria_Alphaproteobacteria*_NA_NA_NA	0.2	0	0	7.2
**47**	*Bacteria_Proteobacteria_Alphaproteobacteria_Rhodospirillales_Rhodospirillaceae*_NA	0.2	0	0	7.3
**48**	*Bilophila*	0.2	0.1	0	1.7
**49**	*Akkermansia*	0.2	0	0	4.3
**50**	*Adlercreutzia*	0.1	0	0	1.6
**51**	*Butyrivibrio*	0.1	0	0	5.6
**52**	*Romboutsia*	0.1	0	0	3.1
**53**	*Intestinimonas*	0.1	0.1	0	1.3
**54**	*Subdoligranulum*	0.1	0	0	4.1
**55**	*Megasphaera*	0.1	0	0	20.8
**56**	*Aestuariispira*	0.1	0	0	6.7
**57**	*Bacteria_Proteobacteria_Betaproteobacteria_Burkholderiales*_NA_NA	0.1	0	0	4.9
**58**	*Haemophilus*	0.1	0	0	4.9
**59**	*Bacteria_Proteobacteria*_NA_NA_NA_NA	0.1	0	0	4.9
**60**	*Bacteria_Tenericutes_Mollicutes*_NA_NA_NA	0.1	0	0	9.1
**61**	*Methanobrevibacter*	0	0	0	2.4
**62**	*Eggerthella*	0	0	0	1.2
**63**	*Bacteria_Actinobacteria_Actinobacteria_Coriobacteriales_Coriobacteriaceae*_NA	0	0	0	0.5
**64**	*Senegalimassilia*	0	0	0	1.3
**65**	*Slackia*	0	0	0	0.6
**66**	*Lactococcus*	0	0	0	0.8
**67**	*Anaerosporobacter*	0	0	0	0.5
**68**	*Filifactor*	0	0	0	0.9
**69**	*Intestinibacter*	0	0	0	0.8

**70**	*Terrisporobacter*	0	0	0	0.3
**71**	*Papillibacter*	0	0	0	1.8
**72**	*Clostridium XVIII*	0	0	0	0.4
**73**	*Turicibacter*	0	0	0	0.7
**74**	*Megamonas*	0	0	0	6.5
**75**	*Victivallis*	0	0	0	0.6
**76**	*Desulfovibrio*	0	0	0	1.7
**77**	*Salmonella*	0	0	0	3.2
